# Supplementary material for: Sources of genomic diversity in the self-fertile plant pathogen, Sclerotinia sclerotiorum, and consequences for resistance breeding
Source: PLoS One. 2022 Feb 7;17(2):e0262891. doi: 10.1371/journal.pone.0262891 (PMC8820597; doi:10.1371/journal.pone.0262891)
Supplement: S2 Table — (DOCX) [file pone.0262891.s002.docx]

S2 Table. Pearson correlation coefficient for pairwise comparison among five disease traits obtained from inoculation of six *B. napus* lines with 17 *S. sclerotiorum* isolates.

| Disease trait | Stem lesion length 7 dai | Stem lesion length 14 dai | Stem lesion length 21 dai | AUDPC | % soft + collapsed lesions |
| --- | --- | --- | --- | --- | --- |
| Lesion length 7 dai | 1.0 | 0.953 | 0.915 | 0.958 | 0.906 |
| Lesion length 14 dai |  | 1.0 | 0.959 | 0.992 | 0.926 |
| Lesion length 21 dai |  |  | 1.0 | 0.985 | 0.904 |
| AUDPC |  |  |  | 1.0 | 0.929 |
| % soft + collapsed lesions |  |  |  |  | 1.0 |
