# Supplementary material for: Sources of genomic diversity in the self-fertile plant pathogen, Sclerotinia sclerotiorum, and consequences for resistance breeding
Source: PLoS One. 2022 Feb 7;17(2):e0262891. doi: 10.1371/journal.pone.0262891 (PMC8820597; doi:10.1371/journal.pone.0262891)
Supplement: S3 Table — (DOCX) [file pone.0262891.s003.docx]

S3 Table. Results from aggressiveness test of 17 *S. sclerotiorum* isolates averaged over six *B. napus* lines.

| Isolate | Location | Sub-populations in Figure 3 | Number of isolates in sub-population | Stem lesion length (mm), Std error, LSD | % soft + collapsed lesions |
| --- | --- | --- | --- | --- | --- |
| AB7 | Mundane, AB | #12 | 3 | 17.4 + 3.2 a | 4.6 |
| #321 | Olds, AB | #17 | 1 | 47.4 + 11.4 b | 24.2 |
| SK44 | Wadena, SK | #16 | 1 | 67.0 + 17.2 bc | 24.5 |
| MB35 | Killarney, MB | #11 | 6 | 69.7 + 16.5 c | 30.2 |
| AB3 | Blackfoot, AB | #6 | 1 | 82.1 + 20.4 cd | 40.2 |
| MB57 | Birtle, MB | #13 | 12 | 82.4 + 9.5 cde | 44.2 |
| SK14 | Melfort, SK | #15 | 12 | 92.4 + 13.6 def | 47.8 |
| MB52 | Shoal Lake, MB | #2 | 14 | 92.5 + 22.1 def | 47.7 |
| SK23 | Alvena, SK | #7 | 5 | 97.0 + 22.3 def | 48.2 |
| MB61 | Westbourne, MB | #9 | 4 | 101.3 + 15.2 defg | 53.1 |
| MB21 | Morton, MB | #10 | 6 | 101.8 + 15.4 efg | 52.5 |
| SK35 | Rouleau, SK | #11 | 6 | 103.0 + 18.2 fg | 45.7 |
| MB51 | Lilyfield, MB | #4 | 22 | 120.3 + 19.4 hg | 64.4 |
| SK45 | Mozart, SK | #5 | 4 | 123.4 + 14.8 h | 66.0 |
| SK38 | Melville, SK | #8 | 3 | 124.7 + 16.1 h | 67.1 |
| AB19 | Stettler, AB | #14 | 13 | 133.8 + 17.0 hi | 68.0 |
| AB29 | Cayley, AB | #1 | 19 | 151.3 + 13.8 i | 73.1 |
| LDS _0.95_ |  |  |  | 19.7 | 11.3 |
