# Supplementary material for: Sources of genomic diversity in the self-fertile plant pathogen, Sclerotinia sclerotiorum, and consequences for resistance breeding
Source: PLoS One. 2022 Feb 7;17(2):e0262891. doi: 10.1371/journal.pone.0262891 (PMC8820597; doi:10.1371/journal.pone.0262891)
Supplement: S4 Table — (DOCX) [file pone.0262891.s004.docx]

S4 Table. Results from aggressiveness test of six *B. napus* lines averaged over 17 *S. sclerotiorum* isolates.

| *B. napus* line | Stem lesion length (mm) + Std error, LSD |
| --- | --- |
| PAK54 | 48.3 ± 3.2 a |
| K22 | 69.8 ± 4.0 b |
| PAK93 | 74.8 ± 4.0 b |
| DC21 | 106.4 ± 6.5 c |
| Tanto | 106.8 ± 4.5 c |
| Topas | 161.2 ± 6.6 d |
| LSD | 11.71 |
