# Supplementary material for: Sources of genomic diversity in the self-fertile plant pathogen, Sclerotinia sclerotiorum, and consequences for resistance breeding
Source: PLoS One. 2022 Feb 7;17(2):e0262891. doi: 10.1371/journal.pone.0262891 (PMC8820597; doi:10.1371/journal.pone.0262891)
Supplement: S5 Table — (DOCX) [file pone.0262891.s005.docx]

S5 Table. Results from testing of 17 *S. sclerotiorum* isolates for aggressiveness on six *B. napus* lines. Plants at full flower were inoculated by attaching a mycelium plug to the main with Para film. The average stem lesion length 21 days after inoculation is shown including the Standard error (see graph in Figure 6).

| Isolate | Topas | Std error | Tanto | Std error | DC21 | Std error | K22 | Std error | PAK93 | Std error | PAK54 | Std error |
| --- | --- | --- | --- | --- | --- | --- | --- | --- | --- | --- | --- | --- |
| AB7 | 35.3 | 10.5 | 19.0 | 2.9 | 10.2 | 1.0 | 10.8 | 1.3 | 16.6 | 2.6 | 12.2 | 0.7 |
| 321 | 54.2 | 8.7 | 59.7 | 18.4 | 42.7 | 11.1 | 72.7 | 18.1 | 37.1 | 10.4 | 17.7 | 1.9 |
| SK44 | 141.2 | 12.4 | 70.6 | 16.0 | 49.0 | 23.4 | 33.9 | 14.9 | 63.0 | 18.6 | 44.2 | 17.8 |
| MB35 | 145.3 | 30.6 | 92.1 | 17.6 | 63.3 | 21.8 | 27.5 | 7.8 | 67.8 | 16.5 | 22.4 | 4.5 |
| AB3 | 109.8 | 28.5 | 85.9 | 20.7 | 110.5 | 26.8 | 102.9 | 19.7 | 38.4 | 11.5 | 44.8 | 15.0 |
| MB57 | 160.0 | 9.9 | 93.4 | 13.2 | 114.3 | 15.6 | 63.8 | 10.8 | 47.2 | 5.2 | 15.9 | 2.2 |
| SK14 | 214.8 | 5.4 | 107.5 | 26.4 | 87.9 | 12.8 | 42.6 | 12.3 | 70.4 | 15.4 | 31.4 | 9.1 |
| MB52 | 182.2 | 12.1 | 118.2 | 23.1 | 78.4 | 31.5 | 58.1 | 30.5 | 71.9 | 15.6 | 46.4 | 19.5 |
| SK23 | 179.6 | 21.8 | 123.0 | 33.9 | 66.3 | 20.0 | 88.2 | 29.9 | 78.0 | 13.6 | 46.7 | 14.4 |
| MB61 | 185.5 | 7.4 | 123.4 | 7.7 | 115.6 | 35.3 | 48.0 | 12.4 | 75.8 | 23.8 | 59.5 | 4.3 |
| MB21 | 164.0 | 19.5 | 120.6 | 18.3 | 124.2 | 22.8 | 46.0 | 8.1 | 121.7 | 8.3 | 34.4 | 15.5 |
| SK35 | 132.1 | 12.9 | 71.8 | 28.2 | 185.4 | 10.4 | 125.8 | 27.1 | 57.1 | 12.3 | 45.7 | 18.4 |
| MB51 | 223.4 | 20.7 | 156.8 | 9.8 | 101.3 | 25.6 | 93.4 | 24.4 | 79.9 | 19.4 | 67.0 | 16.4 |
| SK45 | 203.8 | 16.6 | 140.8 | 22.2 | 128.3 | 15.1 | 78.9 | 13.4 | 103.6 | 2.7 | 84.9 | 18.6 |
| SK38 | 191.2 | 12.3 | 134.3 | 14.9 | 158.5 | 19.5 | 123.3 | 11.8 | 78.6 | 15.4 | 62.3 | 22.4 |
| AB19 | 208.8 | 29.8 | 146.0 | 9.0 | 159.3 | 10.0 | 70.7 | 14.7 | 137.9 | 13.1 | 80.1 | 25.3 |
| AB29 | 209.4 | 11.1 | 153.0 | 26.1 | 212.9 | 13.7 | 100.0 | 5.8 | 126.1 | 15.3 | 106.2 | 10.5 |
