# Supplementary figures and images for: Sources of genomic diversity in the self-fertile plant pathogen, Sclerotinia sclerotiorum, and consequences for resistance breeding
Source: PLoS One. 2022 Feb 7;17(2):e0262891. doi: 10.1371/journal.pone.0262891 (PMC8820597; doi:10.1371/journal.pone.0262891)

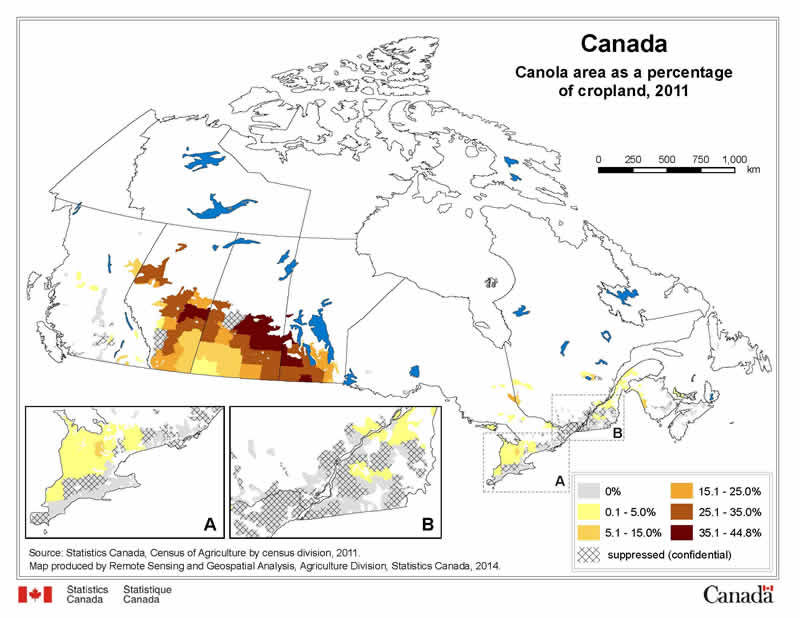

Supplement: S1 Fig — (JPG) [file pone.0262891.s008.jpg]
